# Modified marionette technique for uniportal video-assisted thoracic surgery: a case report

**DOI:** 10.1186/s40792-019-0657-y

**Published:** 2019-06-17

**Authors:** Sumitaka Yamanaka, Kensuke Adachi

**Affiliations:** 1 Department of Thoracic Surgery, Tokyo Shinagawa Hospital, 6-3-22 Higashi-Oi, Shinagawa-ku, Tokyo, 140-8522 Japan; 2 Department of Surgery, Tokyo Metropolitan Health and Hospitals Corporation, Ebara Hospital, Tokyo, Japan

**Keywords:** Video-assisted thoracoscopic surgery, Uniportal, Internal organ retractor

## Abstract

**Background:**

The current state of thoracoscopic technology allows less invasive surgical procedures and requires fewer ports and incisions. Totally video-assisted thoracic surgery with a single port has emerged as the least invasive thoracoscopic approach.

However, uniportal video-assisted thoracic surgery brings about considerable difficulties, necessitating the development of skillful techniques as well as specific surgical devices. In such situations as dense pleural adhesion and anatomical abnormality, it may be more burdensome, necessitating the conversion to conventional multiportal video-assisted thoracic surgery or even to thoracotomy. To troubleshoot these situations, we herein propose the use of additional technique which could support to sustain the confident operative field for uniportal video-assisted thoracic surgery. This procedure also provides the same cosmetic outcomes as uniportal video-assisted thoracic surgery.

**Case presentation:**

A previously healthy, 77-year-old female was referred to our hospital, with a lung adenocarcinoma measuring 28 mm in the right upper lobe. Uniportal video-assisted thoracoscopic surgery was planned to resect the tumor. During operation, we found the incomplete interlobar fissure between the upper and the middle lobe and the abnormal lobulation of the upper lobe. Therefore, the modified marionette technique was introduced to make the procedure safer and easier. This technique proposed herein consists of employing the untethered gripping forceps to retract the lung, not requiring additional traumatic trocars. The postoperative course was uneventful and the patient `was discharged in 1 week after a modified uniportal video-assisted thoracic surgery for the right upper lobectomy.

**Conclusions:**

The modified marionette technique produced sufficient operative views to attain uniportal video-assisted thoracic surgery safely in this case, rendering operative conversion unnecessary.

## Background

Although multiportal video-assisted thoracic surgery (MVATS) has already served as the first-line operative procedure for lung diseases, uniportal video-assisted thoracic surgery (UVATS) has recently emerged as a vital alternative to MVATS due to the advantages of achieving cosmetic benefits and ameliorating the postoperative quality of patients.

The uniportal video-assisted thoracic surgery for pulmonary wedge resection was first reported in 2004 [1]**.** Since then, several publications have discussed extending indications of UVATS. However, UVATS in anatomical lobectomies for pulmonary malignancies still remains controversial because prospective studies comparing it with conventional multiportal video-assisted thoracic surgery have yet to be performed [2].

UVATS might be appropriate for the case in good condition of the lung, whereas such situations as dense pleural adhesion and anatomical abnormality surrounding the tumor impose severe technical burdens.

A good operability of tools based on the confident thoracoscopic views is deemed to be a prerequisite to perform UVATS promptly and accurately.

## Case presentation

A previously healthy, 77-year-old female was referred to our hospital, with a lung adenocarcinoma measuring 28 mm in the right upper lobe. We therefore planned a UVATS to resect the tumor. The patient was placed in the left lateral decubitus position under general anesthesia. Then, we made a 4-cm skin incision for the main port in the sixth intercostal space at the anterior axillary line. A wound retractor (Alexis-xs; Applied Medical, Rancho Santa Margarita, CA) allowed the insertion of a flexible thoracoscope (10 mm in diameter, Olympus Optical Tokyo, Japan), endoscopic autosuturing device (GIA Universal; Covidien, Mansfield, MA or Echelon; Ethicon, Cincinnati, OH), and vessel-sealing device (Ligasure; Covidien) via the main port incision. It also allowed specimen extraction.

**Fig. 1:**
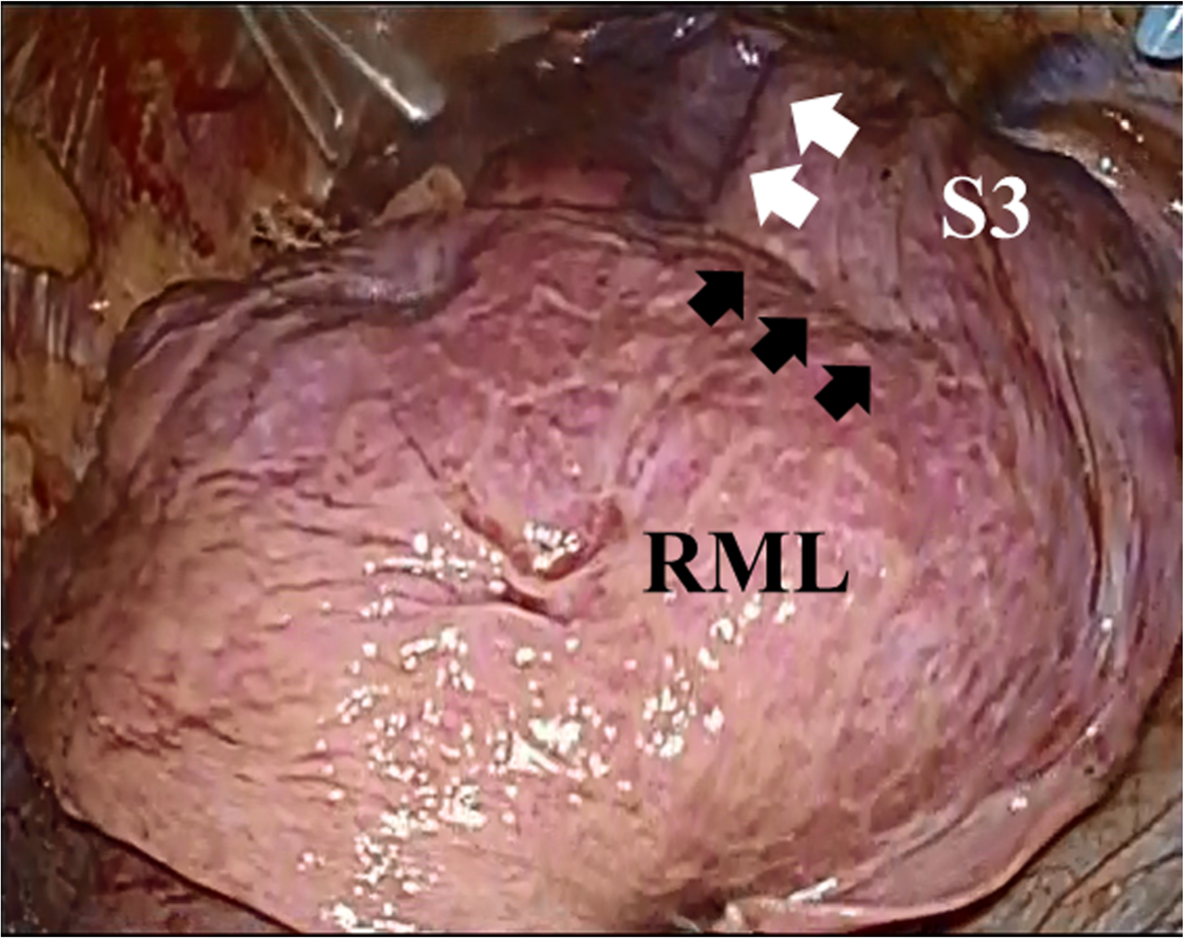
The intraoperative photograph. The incomplete interlobar fissure between the right upper and middle lobe deemed to be complicated to perform UVATS. Black arrows indicate the incomplete interlobar fissure. White arrows indicate the abnormal lobulation of the S3. RML, right middle lobe

**Fig. 2:**
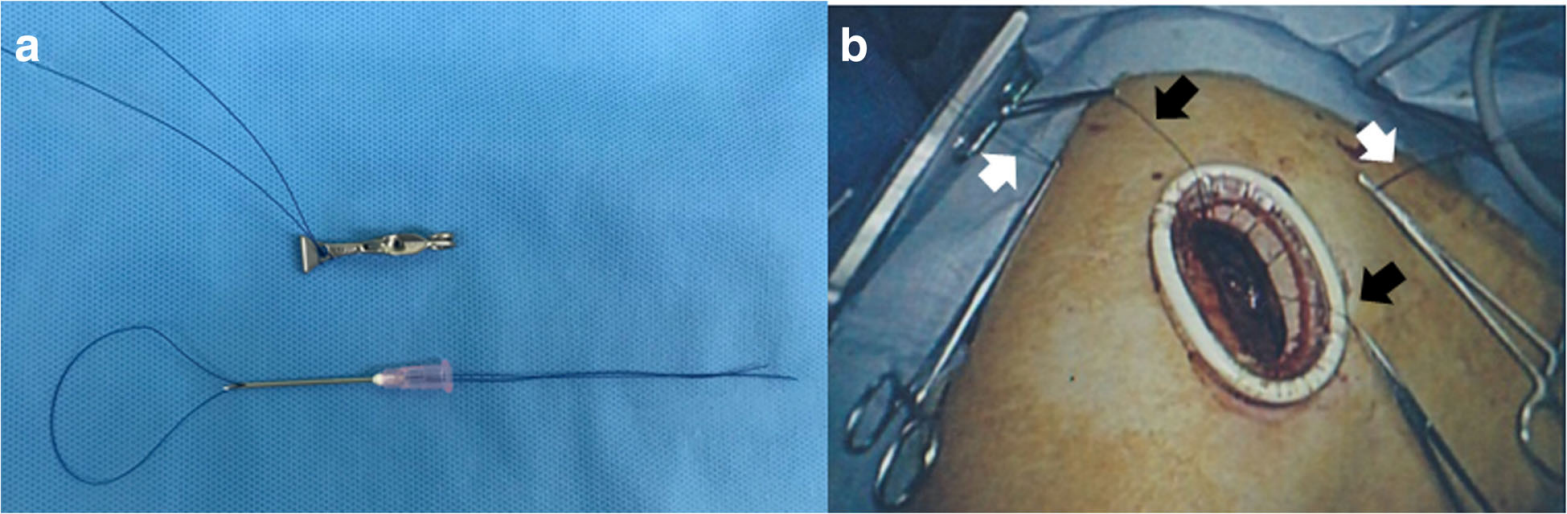
Preparation of the modified marionette technique. **a** The IOR with a 1-0 nylon thread (top) and a looped 1-0 nylon-threaded 18-gauge injection needle (bottom). The 1-0 nylon ligated the IOR through the hole existing at tips of the IOR for proper retraction. **b** Intraoperative view. Mosquito forceps grasp 1-0 nylon threads pulled out from thoracic cavity. White arrows indicate looped 1-0 nylons working as pivots. Black arrows indicate 1-0 nylons attached to the IOR

**Fig. 3:**
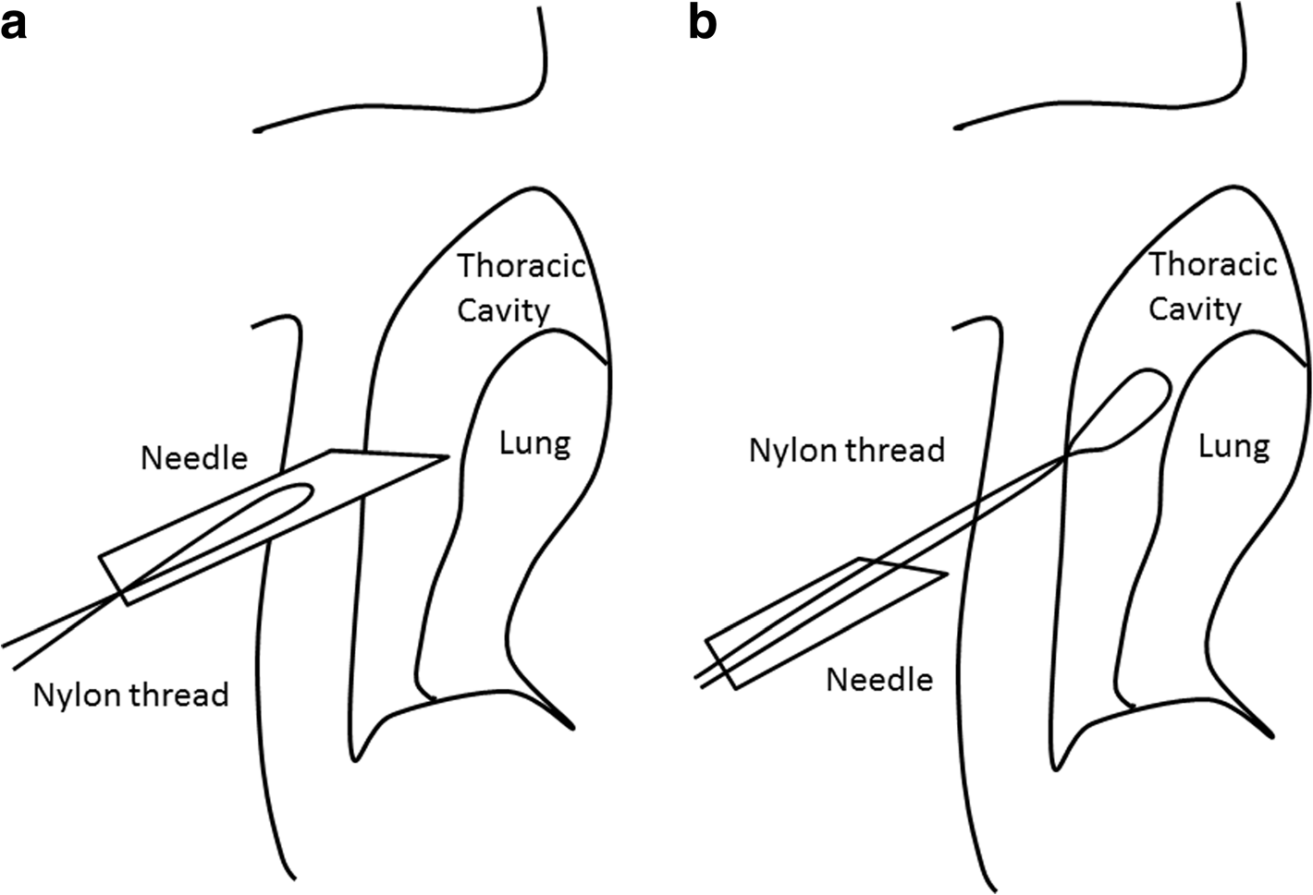
The schema described how to introduce the looped nylon into the thoracic cavity through the needle. **a** The looped nylon thread is kept inside the needle during piecing of the chest wall. **b** The looped nylon is pushed into the thoracic cavity, and then the needle is removed

**Fig. 4:**
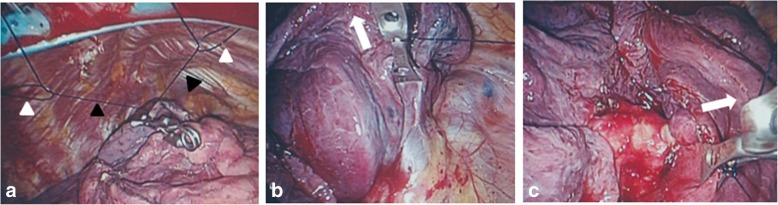
Intraoperative views of the modified marionette technique. **a** The looped 1-0 nylons working as pivots were pierced through the chest wall (white arrow heads). The 1-0 nylons attached to the IOR were drawn out separately form the access port (black arrow heads). **b** When the left nylon attached to the IOR was pulled, the IOR retracted the lung upward toward the left-side looped nylon. White arrows indicate the direction of traction. **c** When the right nylon attached to the IOR was pulled, the IOR retracted the lung upward toward the right-side looped nylon

During operation, we found the incomplete interlobar fissure between the upper and the middle lobe and the abnormal lobulation of the upper lobe. (Fig. 1)*.* Therefore, we carried out the so-called modified marionette technique as follows. First, the Internal organ retractor (IOR; Aesculap, Tuttlingen, Germany) applied with a looped 1-0 nylon thread was inserted into the thoracic cavity by the clip applier (Aesculap). The clip applier also allowed the IOR to grasp the targeted lung parenchyma properly. Second, two sets of looped 1-0 nylon-threaded 18-gauge injection needles were prepared (Fig. 2a). These needles were optimally pierced through the thoracic wall separately (Fig. 3a and b). Third, the both ends of the 1-0 nylon thread attached to IOR were separately pulled out through the looped nylon extruded from the 18-gauge injection needles. Each thread was clamped by mosquito forceps (Fig. 2b). The looped nylon extruded from 18-gauge injection needles worked as a pivot, so that the vector of retraction of the IOR was converted into an ideal direction (Fig. 4). This method gave the sufficient thoracoscopic views to perform surgery with safe and ease. The patient had an uneventful postoperative course and was discharged in 1 week after the operation without any wound complications.

## Discussion

The advent of VATS has improved the postoperative quality of life of patients. Since its introduction, MVATS has become the first-line procedure for anatomical lobectomies of lung neoplasms [3]. Furthermore, UVATS has recently emerged as a viable alternative to MVATS [4–7]. The former indeed has the advantage of reducing the likelihood of postoperative wound complications but is sometimes inferior to the latter in terms of perioperative outcomes [4–6].

Despite advantages of UVATS, the single-port access poses several dilemmas. It not only requires specific thoracoscopic devices, but also imposes technical burdens, i.e., the difficulty of applying the techniques in a thoracoscopy including the operability of the surgical instruments and/or the difficulty to sustain the confident operative field [8].

The “marionette trick” was first introduced in laparoscopic surgery in 2003 [9]. This technique means traction after suturing the target mass through the abdominal wall, rendering insertion of additional trocars unnecessary. Another merit of this trick includes changing the traction sites easily according to the intraoperative circumstances; however, we are unable to apply this traumatic technique to lung surgery.

The IOR has been recently discussed in single-port laparoscopic surgery [8, 10, 11]. The IOR has untethered gripping forceps to permit holding parenchymal organs atraumatically.

Modified marionette technique using IOR for Laparoscopic Colorectal Surgery was reported in 2017 [8]. Our method described in this report was basically the same, but it required some improvement to apply the technique to thoracic surgery. The site of the attachment of a nylon thread and the IOR was devised as shown in Fig. 2a, because of the narrowness of the thoracic cavity compared with the abdominal cavity using pneumoperitonium.

Furthermore, two traction threads with the IOR led to the two-dimensional mobilization of the lung. This modification produces a good operability based on the clean thoracoscopic views enough to perform UVATS promptly and accurately.

This case had the incomplete interlobar fissure between the upper and the middle lobe and the abnormal lobulation of the upper lobe. In such a case, it is important to ascertain the running direction of pulmonary veins at the hilum of the lung as well as dissecting the interlobar fissure. This maneuver enabled us to mobilize the lung efficiently, perform countertracion of the tissues, and maintain the confident thoracoscopic view without insertion of additional trocars.

The magnetic-based device for tissue retraction had recently reported [12]. However, the magnetic system has not spread yet, and it was inferior to our method regarding the feasibility and the cost-effectiveness.

The skillful and expert surgeons might insist that the special devices were not necessary to perform UVATS. However, this system can be salutary for trainees and beneficial for the cases in poor condition of the lung. The valuable devices without depending on individual techniques might be necessary for the paradigm shift of UVATS procedure.

## Conclusions

The modified marionette technique produced sufficient operative views to attain uniportal video-assisted thoracic surgery safely in this case. This method can be achieved at low cost and be readily performed at centers currently performing VATS. However, collection of patient data is necessary to evaluate clinical benefits in this method.

## Data Availability

Data sharing not applicable to this article as no datasets were generated or analyzed during the current study.

## Abbreviations

IOR: Internal organ retractor
MVATS: Multiportal video-assisted thoracic surgery
UVATS: Uniportal video-assisted thoracic surgery
